# Analysis of the difference in the perinatal mortality rate between white-collar and blue-collar workers in Japan, 1995-2015

**DOI:** 10.4178/epih.e2020069

**Published:** 2020-11-24

**Authors:** Tasuku Okui

**Affiliations:** Medical Information Center, Kyushu University Hospital, Fukuoka, Japan

**Keywords:** Japan, Social class, Perinatal mortality, Middle aged

## Abstract

**OBJECTIVES:**

This study investigated differences in the perinatal mortality rate between white-collar and blue-collar workers.

**METHODS:**

Data from the “Report of Vital Statistics: Occupational and Industrial Aspects” in Japan covering the period from 1995 to 2015 were used. Five-year maternal age groups from 15-19 years to 45-49 years were analyzed according to work type, and the perinatal mortality rate for each age group and the age-standardized perinatal mortality rate according to maternal age were calculated in each analyzed year. A Bayesian age-period-cohort analysis was used to estimate age, period, and cohort effects for the perinatal mortality rate according to work type. Moreover, the perinatal mortality rate ratios between types of workers were estimated for each age group, period, and cohort.

**RESULTS:**

The estimated perinatal mortality rate ratios of blue-collar to white-collar workers were above 1 in most of the age groups and cohorts. The age effect for the perinatal mortality rate among white-collar workers was the largest in the 15-year to 19-year age group, whereas that among blue-collar workers was the largest in the 45-year to 49-year age group. Furthermore, the estimated perinatal rate ratio between white-collar and blue-collar workers tended to increase with maternal age. The magnitude of the decrease of the cohort effects on the perinatal mortality rate was rather larger in blue-collar workers in the cohorts born between 1946-1950 and 1996-2000.

**CONCLUSIONS:**

The magnitude of the disparity markedly increased with maternal age. Thus, middle-aged blue-collar workers need more prenatal care and preventive measures for perinatal mortality than white-collar workers.

## INTRODUCTION

Japan is known for its favorable perinatal outcomes by current global standards [1]. Fetal death rates and perinatal mortality rates have been used as representative indicators of perinatal health in Japan, with their results being published annually in the Vital Statistics. Although the perinatal mortality rate per 1,000 births was over 20 in 1979, this rate subsequently decreased to below 4 in 2016 [1]. Moreover, studies have shown that fetal death rates have decreased since the 1950s, with fetal death rates due to spontaneous and induced abortions also decreasing over the years [1].

Nonetheless, perinatal outcomes, such as stillbirth or perinatal mortality, are known to be influenced by the socioeconomic status (SES) of mothers or households [2-5]. Indeed, some studies, albeit few, have recently investigated associations between SES and perinatal outcomes using the Vital Statistics in Japan [3,4]. One such study showed that jobless households had higher perinatal mortality and abortion rates than other households in Japan [4]. Another epidemiological study found that small-for-gestational-age births were associated with maternal SES in Japan [5]. Although the reason for the association between SES and perinatal outcomes has yet to be determined [2,5], one possible explanation could be the effect of lower income or educational background on psychological stress in mothers [5]. Disparities among occupational classes, as an indicator of SES, have often been the focus of public health research, particularly differences between white-collar and blue-collar workers [6-8]. Disparities between white-collar and blue-collar workers have also been highlighted in perinatal health studies [8,9]. For instance, a Finnish study showed disparities in small-for-gestational-age birth rates, perinatal mortality rates, and low birthweight rates between upper white-collar and blue-collar workers [8]. Another study using Vital Statistics data in Japan showed that perinatal mortality rates and fetal death rates varied according to maternal occupation [10]. However, no study has yet analyzed differences in perinatal outcomes among occupational classes, such as between white-collar and blue-collar workers. Japanese studies have revealed health differences between white-collar and blue-collar workers for some diseases [6,7], suggesting the possibility that perinatal outcomes may vary according to the occupational classes. Moreover, the magnitude of differences in perinatal outcomes among occupational classes has been considered to vary according to age, period, and cohort, although no Japanese study has yet analyzed differences in perinatal outcomes while considering differences according to such factors.

Age-period-cohort (APC) analysis is a useful method for analyzing trends in perinatal outcomes [11-13] and has often been used for analyzing disparities in disease-related mortality according to SES. Moreover, APC analysis enables researchers to determine differences in outcome trends according to SES over age, period, and cohort. For instance, previous perinatal health studies utilizing APC analysis investigated differences in small-for-gestational age rates and infant mortality rates between Black and White populations to assess the structures of disparities [11,13]. Considering that trends in perinatal outcomes might differ according to occupational classes in Japan, APC analysis may facilitate a better understanding of actual conditions affecting such differences. The current study therefore analyzed differences in perinatal mortality rates between white-collar and blue-collar workers using APC analysis.

## MATERIALS AND METHODS

### Data

This study focused on the perinatal mortality rate. Perinatal mortality is defined as fetal deaths after the 22nd week of pregnancy and neonatal deaths within 1 week after birth [1]. As such, the perinatal mortality rate is defined as the number of perinatal deaths per the sum of the total number of births and perinatal deaths. Although “rate” in epidemiology is a measure of the frequency with which an event occurs in a defined population over a specified period, we use the term “rate” herein to indicate an annual rate, following common practice in previous studies [11-13].

This study utilized data from the “Report of Vital Statistics: Occupational and Industrial Aspects” in Japan covering 1995 to 2015 [14], which have been used to analyze mortality according to occupational categories [15,16]. This report is published every 5 years and incorporates data from Vital Statistics according to occupational categories. Occupational categories changed twice during the relevant periods. Accordingly, 9 occupational categories were available in 1995, including administrative and managerial; professional; clerical; sales; services; security; agriculture, forestry, and fishing; and craft, mining, manufacturing, construction, and labor work. In 2000 and 2005, craft, mining, manufacturing, construction, and labor work were removed from the occupational categories, and 2 categories (transport and production process and labor work) were subsequently added. In 2010 and 2015, production process and labor work were removed from the occupational categories, and 3 categories (production process; construction and mining; and carrying, cleaning, and packaging) were subsequently added. Previous studies have classified occupational categories into white-collar and blue-collar workers, although the precise definitions have varied across different studies [17,18]. As such, white-collar workers were herein defined using the 3 categories generally used to describe white-collar workers (i.e., administrative and managerial, professional, and clerical). Blue-collar workers were defined using categories often associated with blue-collar workers (i.e., agriculture, forestry, and fishing; craft, mining, manufacturing, construction, and labor work; transport; production process and labor work; production process; construction and mining; and carrying, cleaning, and packaging).

### Statistical analysis

Five-year maternal age groups from 15-19 years to 45-49 years were used for the analysis, with the oldest birth cohort being 45-49 years of age in 1995 (those born in 1946-1950) and the youngest cohort being 15-19 years of age in 2015 (those born in 1996-2000). Thereafter, the perinatal mortality rate for each maternal age group and year was calculated according to the type of worker. Moreover, the age-standardized perinatal mortality rate according to maternal age was calculated using the total population ratio in 1995 as the standard population. Subsequently, a Bayesian APC model whose outcome variable followed a Poisson distribution was fitted to the data [19]. The sum of each age, period, and cohort effect was restricted to zero, and a first-order random-walk was used as a Bayesian prior for each effect to identify each age, period, and cohort effect [20]. The APC model for perinatal mortality rate was fitted according to each type of worker, and age, period, and cohort effects were presented. Moreover, the estimated perinatal mortality rates were calculated using the intercept and each effect in the APC model for each type of worker. Thereafter, the estimated perinatal mortality rate ratios for blue-collar workers compared with white-collar workers were calculated for each age group, period, and cohort. To confirm goodness-of-fit in the APC model, we also analyzed the data by an age model, a period model, a cohort model, an age-period model, an age-cohort model, and a period-cohort model. The deviance information criterion was calculated for each model, and the goodness-of-fit of all models was compared. All statistical analyses were conducted using R version 3.6.3 (https://cran.r-project.org/bin/windows/base/old/3.6.3/), whereas the estimation of the APC model was conducted using Stan (http://mc-stan.org). Although the models were fitted according to each type of worker, the data for white-collar and blue-collar workers were simultaneously modelled in Stan. Since publicly available data were used, ethical approval was not needed for this study.

### Ethics statement

The data that are publicly available were used in this study, and an institutional review board approval was not needed in this study.

## RESULTS

Table 1 shows the perinatal mortality and total births for each age group, period, and type of worker. The numbers of births and perinatal mortality were somewhat smaller in the age groups of 15-19 years and 45-49 years in both types of workers.

Table 2 shows the perinatal mortality rate for each age group, as well as the age-standardized perinatal mortality rate according to maternal age per 1,000 births for each period and type of worker. Perinatal mortality rates tended to decrease from 1995 to 2015 regardless of maternal age and type of worker. Moreover, the perinatal mortality rates of white-collar workers tended to be lower than those of blue-collar workers among individuals aged > 25 years regardless of period, whereas blue-collar workers tended to have lower perinatal mortality rates at younger ages.

Table 3 shows the result of the deviance information criterion. The age-cohort model had the lowest value among the models. Therefore, we presented the results of the age-cohort model in Table 4.

Table 4 details the results of the age-cohort model according to the type of workers. Although the age effect for perinatal mortality rate among white-collar workers was largest in the 15-year to 19-year age group, that among blue-collar workers was largest in the 45-year to 49-year age group. The birth cohort effects decreased over cohorts, respectively, in both types of workers, and the magnitude of the decrease in the cohort effect in the cohorts born between 194-1950 and 1996-2000 was larger in blue-collar workers.

Table 5 shows the perinatal mortality rate ratios between blue-collar and white-collar workers according to age and cohort. The rate ratios were above 1 most of the time regardless of age and cohort. Although the rate ratio was below 1 in younger ages, it increased with age, and a statistically significant difference was observed from the age groups of 25-29 years to 45-49 years. In contrast, the rate ratios particularly decreased in the cohorts born between 1946-1950 and 1956-1960, and a statistically significant difference was not observed in most of the birth cohorts.

## DISCUSSION

This is the first study investigating the difference in perinatal outcomes between blue-collar and white-collar workers in Japan. The estimated perinatal mortality rate ratios between blue-collar and white-collar workers were above 1 in most of the age groups and cohorts, and the magnitude of the disparity depended on age and cohort. The possible reasons for these trends are discussed below.

Age effects on the perinatal mortality rate tended to increase in older blue-collar and white-collar workers, as well as in younger white-collar workers. Previous studies have shown that maternal age was a major risk factor for stillbirth or perinatal mortality [21,22], and the results of the current study support such findings. However, blue-collar workers exhibited a greater increase in maternal age effects than white-collar workers, and the perinatal mortality rate ratios tended to increase with age. One possible reason for this could be differences in SES between both types of workers depending on maternal age. According to the Basic Survey on Wage Structure in Japan [23], a subtle difference in wages was observed between both types of workers in their early 20s, with the wage disparity subsequently enlarging as age increased. Therefore, differences in the extent of perinatal mortality disparity according to maternal age might have been due to differences in SES. Another reason for the high perinatal mortality rates in older women could be an increased risk of pregnancy complications with maternal age. Accordingly, pre-eclampsia or gestational hypertension is a typical pregnancy complication that can lead to stillbirth, the incidence rate of which is related to both maternal age and maternal SES [24-26]. Therefore, trends in perinatal mortality rate ratios according to age in both types of workers might suggest that the effects of SES differences on maternal complications tend to become more evident with age. Moreover, psychological stress is considered to be a possible factor for poor perinatal outcomes in women with low SES in Japan [5]. In Japan, the prevalence of mental illness among women particularly increases in middle age [27], and worse psychological status might have contributed to a higher perinatal mortality rate for blue-collar workers.

In contrast, cohort effects decreased over cohorts in both types of workers, with the extent of the decrease being larger for blue-collar workers. As shown in Table 2, the extent of the decrease in perinatal mortality rates for blue-collar workers in age groups of over 40 years old was larger compared with that in other age groups, resulting in a larger decrease in the cohort effect. Cohort effects are considered to indicate changes in perinatal mortality rates according to maternal factors that can change depending on birth cohorts, and archetypal causes of perinatal mortality related to maternal factors include pregnancy complications, such as preeclampsia or gestational hypertension. Educational qualification is a maternal factor that varies depending on the maternal birth cohort, and is associated with poor perinatal outcomes in Japan [5]. The level of education has risen with each birth cohort in Japan [28], and it is possible that the number of blue-collar workers with low educational levels decreased across the birth cohorts. Therefore, perinatal mortality caused by maternal factors such as a low educational level or maternal complications may have decreased over the maternal cohorts, particularly in blue-collar workers. Smoking status is another predictor of perinatal mortality [26], and its prevalence changed across the cohorts [29]. In Japan, the prevalence of smoking among manual workers is higher than that of non-manual workers [29], and the prevalence of smoking in both types of workers has decreased over time. However, the difference in smoking prevalence between manual and non-manual workers increased from 2001 to 2016 [29]. Therefore, factors other than smoking prevalence are thought to be associated with the cohort effects.

Some limitations of the current study are worth noting. Considering that APC analysis is descriptive in nature, accurate explanations for trends in indicators cannot be ascertained. Further epidemiological studies that consider SES or occupational categories are needed in order to clarify the underlying causes. Another limitation is that although white- and blue-collar workers were classified based on occupational categories, the content of blue-collar work varies depending on the definition. In particular, not all blue-collar workers as defined in this study necessarily engaged in manual labor.

In conclusion, we revealed differences in perinatal mortality rates between white-collar and blue-collar workers in Japan. The estimated perinatal mortality rate ratios of blue-collar to white-collar workers were above 1 in most of the age groups and cohorts, and the estimated perinatal mortality rate ratio between blue-collar and white-collar workers increased with maternal age. In contrast, the magnitude of the decrease in the cohort effect was rather larger in blue-collar workers in the cohorts born between 1946-1950 and 1996-2000. The degree of the disparity particularly increased with maternal age; thus, middle-aged blue-collar workers need more prenatal care and preventive measures against perinatal mortality than white-collar workers.

## Figures and Tables

**Table 1.** Perinatal mortality and total births for each age group, period, and type of worker

Variables	Maternal age, yr						
	15-19	20-24	25-29	30-34	35-39	40-44	45-49
No. of births							
White-collar							
1995	349	17,223	68,742	61,697	18,846	2,201	66
2000	282	14,179	69,122	66,455	24,755	3,005	81
2005	201	9,280	51,992	75,780	31,073	4,412	124
2010	164	9,651	59,709	91,809	55,301	9,111	210
2015	182	9,375	68,660	114,390	74,888	17,652	445
Blue-collar							
1995	230	5,185	13,048	9,471	2,754	366	21
2000	225	3,341	9,207	7,381	2,551	378	18
2005	118	2,398	6,011	6,798	2,765	424	15
2010	130	2,737	6,543	7,410	4,268	799	10
2015	140	2,644	7,373	8,612	5,545	1,309	23
No. of perinatal deaths							
White-collar							
1995	7	159	421	383	172	27	2
2000	6	104	349	334	135	37	2
2005	4	55	207	315	142	30	0
2010	2	52	172	334	226	74	1
2015	0	33	207	316	254	91	3
Blue-collar							
1995	5	45	93	73	26	16	4
2000	2	18	62	46	20	9	0
2005	0	11	31	35	11	9	0
2010	2	9	38	31	39	8	0
2015	0	6	36	29	31	11	1

**Table 2.** The perinatal mortality rate for each age group, as well as the age-standardized perinatal mortality rate according to maternal age per 1,000 births for each period and type of worker

Type of worker	Maternal age, yr							Age-standardized rate
	15-19	20-24	25-29	30-34	35-39	40-44	45-49	
White-collar								
1995	19.7	9.1	6.1	6.2	9.0	12.1	29.4	6.9
2000	20.8	7.3	5.0	5.0	5.4	12.2	24.1	5.4
2005	19.5	5.9	4.0	4.1	4.5	6.8	0.0	4.4
2010	12.0	5.4	2.9	3.6	4.1	8.1	4.7	3.7
2015	0.0	3.5	3.0	2.8	3.4	5.1	6.7	3.0
Blue-collar								
1995	21.3	8.6	7.1	7.6	9.4	41.9	160.0	8.3
2000	8.8	5.4	6.7	6.2	7.8	23.3	0.0	6.7
2005	0.0	4.6	5.1	5.1	4.0	20.8	0.0	5.1
2010	15.2	3.3	5.8	4.2	9.1	9.9	0.0	5.4
2015	0.0	2.3	4.9	3.4	5.6	8.3	41.7	4.2

**Table 3.** The result of goodness of fit of the models

Models	Degree of freedom	Deviance information criterion
Age	56	460.3
Period	60	468.0
Cohort	48	465.0
Age-period	48	438.8
Age-cohort	36	435.3
Period-cohort	40	461.6
Age-period-cohort	28	438.6

**Table 4.** The result of the age-cohort model according to the type of worker

Effects	Perinatal mortality rate ratio (95% credible interval)	
	White-collar	Blue-collar
Maternal age (yr)		
15-19	3.22 (2.05, 4.95)	1.36 (0.79, 2.43)
20-24	1.39 (1.20, 1.61)	0.84 (0.63, 1.09)
25-29	0.77 (0.67, 0.87)	0.83 (0.65, 1.03)
30-34	0.67 (0.59, 0.76)	0.64 (0.50, 0.82)
35-39	0.62 (0.55, 0.71)	0.76 (0.59, 0.97)
40-44	0.85 (0.73, 1.00)	1.28 (0.98, 1.68)
45-49	0.82 (0.47, 1.32)	1.69 (0.86, 3.68)
Maternal birth cohort		
1946-1950	2.53 (1.39, 5.08)	4.29 (1.75, 13.52)
1951-1955	2.28 (1.70, 3.05)	2.90 (1.75, 4.94)
1956-1960	2.16 (1.81, 2.59)	1.57 (1.12, 2.15)
1961-1965	1.40 (1.21, 1.63)	1.29 (1.00, 1.66)
1966-1970	1.21 (1.06, 1.38)	0.95 (0.75, 1.20)
1971-1975	0.99 (0.87, 1.13)	0.98 (0.79, 1.22)
1976-1980	0.83 (0.73, 0.95)	0.76 (0.59, 0.96)
1981-1985	0.63 (0.54, 0.73)	0.66 (0.50, 0.86)
1986-1990	0.60 (0.51, 0.71)	0.57 (0.41, 0.76)
1991-1995	0.42 (0.31, 0.55)	0.41 (0.24, 0.66)
1996-2000	0.36 (0.17, 0.64)	0.36 (0.11, 0.81)

**Table 5.** Perinatal mortality rate ratios between blue- and white-collar workers according to age and cohort

Variables	Perinatal mortality rate ratio (95% credible interval)
Maternal age (yr)	
15-19	0.59 (0.26, 1.37)
20-24	0.84 (0.62, 1.14)
25-29	1.50 (1.17, 1.94)
30-34	1.34 (1.03, 1.76)
35-39	1.69 (1.28, 2.24)
40-44	2.09 (1.45, 2.97)
45-49	2.87 (1.09, 8.44)
Maternal birth cohort	
1946-1950	2.36 (0.70, 9.00)
1951-1955	1.77 (0.94, 3.35)
1956-1960	1.01 (0.68, 1.50)
1961-1965	1.28 (0.93, 1.76)
1966-1970	1.10 (0.83, 1.46)
1971-1975	1.38 (1.06, 1.80)
1976-1980	1.27 (0.95, 1.70)
1981-1985	1.47 (1.06, 2.02)
1986-1990	1.31 (0.89, 1.91)
1991-1995	1.36 (0.67, 2.61)
1996-2000	1.38 (0.34, 4.68)
